# Dissociated Neural Processing for Decisions in Managers and Non-Managers

**DOI:** 10.1371/journal.pone.0043537

**Published:** 2012-08-22

**Authors:** Svenja Caspers, Stefan Heim, Marc G. Lucas, Egon Stephan, Lorenz Fischer, Katrin Amunts, Karl Zilles

**Affiliations:** 1 Institute of Neuroscience and Medicine, Research Centre Jülich, Jülich, Germany; 2 JARA-BRAIN, Jülich-Aachen Research Alliance, Jülich, Germany; 3 Department of Psychiatry, Psychotherapy, and Psychosomatics, Rheinisch-Westfälische Technische Hochschule Aachen, Aachen, Germany; 4 Section Neurological Cognition Research, Department of Neurology, Rheinisch-Westfälische Technische Hochschule Aachen, Aachen, Germany; 5 Department of Psychology, University of Cologne, Cologne, Germany; 6 Department of Business Studies – Leadership and Organization, FernUniversität Hagen, Hagen, Germany; 7 Institute of Economic and Social Psychology, University of Cologne, Cologne, Germany; 8 C. and O. Vogt Institute for Brain Research, Heinrich-Heine-University Düsseldorf, Düsseldorf, Germany; Bellvitge Biomedical Research Institute-IDIBELL, Spain

## Abstract

Functional neuroimaging studies of decision-making so far mainly focused on decisions under uncertainty or negotiation with other persons. Dual process theory assumes that, in such situations, decision making relies on either a rapid intuitive, automated or a slower rational processing system. However, it still remains elusive how personality factors or professional requirements might modulate the decision process and the underlying neural mechanisms. Since decision making is a key task of managers, we hypothesized that managers, facing higher pressure for frequent and rapid decisions than non-managers, prefer the heuristic, automated decision strategy in contrast to non-managers. Such different strategies may, in turn, rely on different neural systems. We tested managers and non-managers in a functional magnetic resonance imaging study using a forced-choice paradigm on word-pairs. Managers showed subcortical activation in the head of the caudate nucleus, and reduced hemodynamic response within the cortex. In contrast, non-managers revealed the opposite pattern. With the head of the caudate nucleus being an initiating component for process automation, these results supported the initial hypothesis, hinting at automation during decisions in managers. More generally, the findings reveal how different professional requirements might modulate cognitive decision processing.

## Introduction

Decision theories postulate bounded rationality to be the basic problem in decision-making in a complex environment, assuming a trade-off between costs and benefits for or against extensive decision making in situations where information is typically incomplete and cognitive resources are limited [1], [2]. Thus, it is thought that two options might exist to deal with this problem and find a resource-sparing solution: (i) relying on an optimization strategy under given constraints [3]; or (ii) basing decisions on heuristics, i.e. over-learned habits and hard-wired solutions [1], [4]. Both approaches would support the idea of dual processing theories which distinguish between two systems: an automated, intuitive processing system which is typically involved in fulfilling the heuristic approach, and an analytic reasoning system which alone might be overwhelmed when reaching its analytic processing capacity [5], [6]. Usage of one or the other system depends on influencing factors such as saliency of incoming stimuli and availability of resources [7], [8]. It was particularly argued that especially experts make use of the automated processing system by acquiring respective gist knowledge, whereas novices would need to rely on analytic reasoning instead. The automated system comes in handy in situations with equably repetitive decisions which can easily be based on known rules or categories while it might be prone to errors in novel situations [9], [10].

With respect to decision making, managers may be regarded as experts since their job, independent of hierarchy, requires them to decide and to answer for this decision. In its basic ideas, this is independent of the success of the manager since it is just a basic job requirement. Overall, the need for fast decisions in the work environment increased, aggravating the problem of incomplete information. It was therefore assumed that managers as opposed to non-managers must have access to respective strategies to adequately deal with this situation. The manager should be able to make fast choices with limited information and limited cognitive resources, but at the same time be as accurate as possible, e.g. by relying on simplified mechanisms and heuristics [6], [11]. Thus, it was thought that managers might often make use of the non-rational, intuitive processing system [12]–[17]. Such processing approach could be learned [18]–[19] and might develop by repetitive confrontation with the same kind of decisions [20]. However, it is still unknown if this strategy of managers has its neurofunctional correlate in the recruitment of other neural networks than in non-managers. It has to be noted that this kind of decision only encompasses one type of decisions required in daily work life. Depending on the situation, decisions might be based on a profound analysis of the complete situation and all available background information. In the present study, we focused on decisions which can be based on rules or heuristics due to their equable repetitiveness.

From a neuroscientific perspective, ample evidence supports the view of such bipartite processing systems [21]. The two systems were described with differing attributes: deliberative vs. affective system [22], long-run vs. short-run player [23], controlled vs. automatic [24], or controlled vs. emotional [25]. Irrespective of the respective label, it was assumed that for decision-making both systems interact: the affective or automatic system was assumed to be the standard operating system, being only overruled by the control system if necessary (e.g. bad outcome, suboptimal decision processes). Depending on the task, different cortical areas are involved in either of these systems. Typically, areas of the lateral and medial prefrontal cortex were found to be activated during decision-making tasks [35], [36]. Additional activations are found in occipital, parietal, and temporal areas for stimulus processing (e.g. visually presented stimuli) and for preparation for decision-making [28]–[31]. For categorization of stimuli, the relevance of a loop between prefrontal cortex and the basal ganglia was stressed [32]–[34]. Nevertheless, it still remains elusive how other factors might influence the use of either system, especially with respect to adaptation to new situations [21].

**Figure 1:**
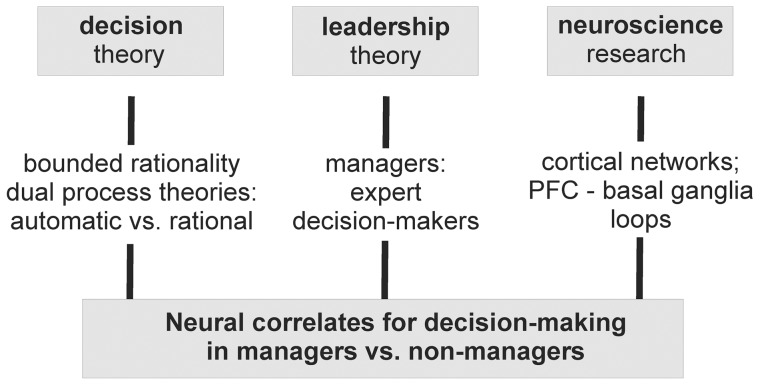
Schematic depiction of the main theory strands which contributed to the study design.

In the present functional magnetic resonance imaging (fMRI) study, we thus analyzed how neural processing mechanisms for decision-making might be influenced by the expertise of decision-making. The line of argumentation is based on theories of social psychology, decision making, and leadership, as well as knowledge about neural processes during decision making (Fig. 1). We tested managers (as expert decision-makers) and non-managers on a repetitive abstract decision-making paradigm on word pairs. Such a paradigm assured that both groups had comparable starting conditions, i.e. a new situation which does not typically occur in daily life.

## Results and Discussion

### Behavioural analysis

Fast decision-making was assessed in managers and non-managers using a repetitive, forced-choice paradigm on 540 word pairs. Words represented basic moral values of either individualistic or collectivistic category [35]. In this abstract decision-making setting (as opposed to real-world complex decision scenarios) subjects were instructed to spontaneously select the most appealing word in each word pair. Based on each person's own moral value orientation, he or she would preferentially select words of one or the other category [35], [36]. Thus, there were no correct or wrong answers. The rule for a decision had to be found out by the subjects themselves. This experimental situation resembles decisions in every-day life where categorization rules are not always externally set, but need to be internally generated by the subjects to deal with incoming information.

The majority of the managers (35 out of 44) preferentially chose individualistic words (individualistic/collectivistic: 304±20/228±24, t-test: *T_1,68_* = 14.40, *P*<0.001). To account for this behavioural impact, we focused further analysis on these individualistically oriented managers, since the neurobiological correlate might differ depending on the moral value orientation of a person [35]. As a control group, 35 non-managers matched for age and gender, and with comparable preferential choices for individualistic words (individualistic/collectivistic: 278±21/254±20, t-test: *T_1,68_* = 4.83, *P*<0.001), were recruited. Managers and non-managers did not differ with regard to IQ (managers/non-managers: 127±11/124±12, t-test: *T_1,68_* = −1.18 *P* = 0.24) or educational level (level of school education: *U_1,68_* = 0.1429, *P* = 0.8674; level of professional training: *U_1,68_* = 0.0857, *P* = 0.9995; Kolmogorov-Smirnov-Test [28]).

**Figure 2:**
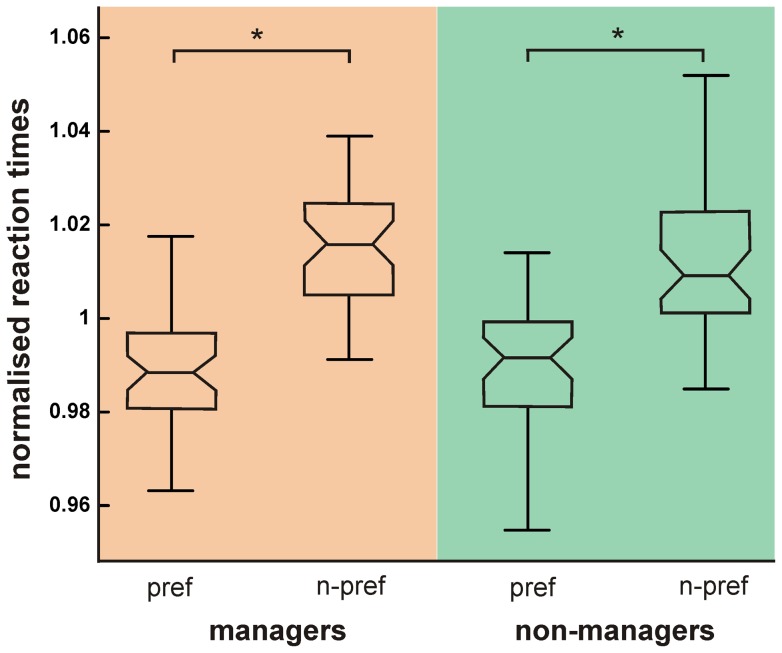
Response time (RT) analysis of managers and non-managers. Box plots showing mean normalized RTs with percentiles (normalized by each subject’s mean RT to account for considerable intersubject variability) for decisions for preferred and non-preferred value words. Asterisks mark significant RT differences. Note the interaction effect in favour of the managers (see text).

In order to test for behavioural effects which might provide a first hint on differential decision processing in managers as compared to non-managers, there were two available data sets: (i) the number of choices for either word category; (ii) the reaction times. Comparing the choice counts between both groups showed a significant interaction effect between word category (individualistic vs. collectivistic) and group (managers vs. non-managers): The difference between choices of individualistic and collectivistic words is larger in managers than in non-managers (*F_1,136_* = 51.79, *P*<0.001, two-way ANOVA). Analysis on the single word level revealed preponderant choices for individualistic words such as ‘success’, ‘autonomy’, ‘competence’, ‘performance’, ‘risk-taking’, ‘determination’, ‘respect’ or ‘challenge’ for the managers as compared to the non-managers. Analysis of the response times (RT) during decision-making revealed significantly shorter RTs for preferred (individualistic) vs. non-preferred (collectivistic) word category (*F_1,136_* = 60.63, *P*<0.001, two-way ANOVA; Fig. 2)). This effect was mainly driven by the managers as revealed by a significant interaction effect (*F_1,136_* = 16.18, *P*<0.001, two-way ANOVA): managers had a more pronounced RT difference between preferred and non-preferred choices than the non-managers.

Both these analyses point in the same direction: managers seem to have been able to more clearly sort out the words of the preferred category, with regard to the absolute number of choices as well as to the speed of processing. It is important to note that managers did not generally make faster decisions, but were particularly faster when deciding for a more familiar, preferred category. Since the task was to select the most appealing word in each word pair, the managers might have found a more efficient way of sorting the presented words. This might have enabled them to faster decide for their preferred category. Taken together, these behavioural findings provide first support for the hypothesis that managers might rely on a different decision processing system. This could be a clear heuristic which allows them to clearly categorize the presented words and to extract the preferred ones, indicating at efficient processing of the presented stimuli.

### Neural correlates for decision-making in managers vs. non-managers

**Figure 3:**
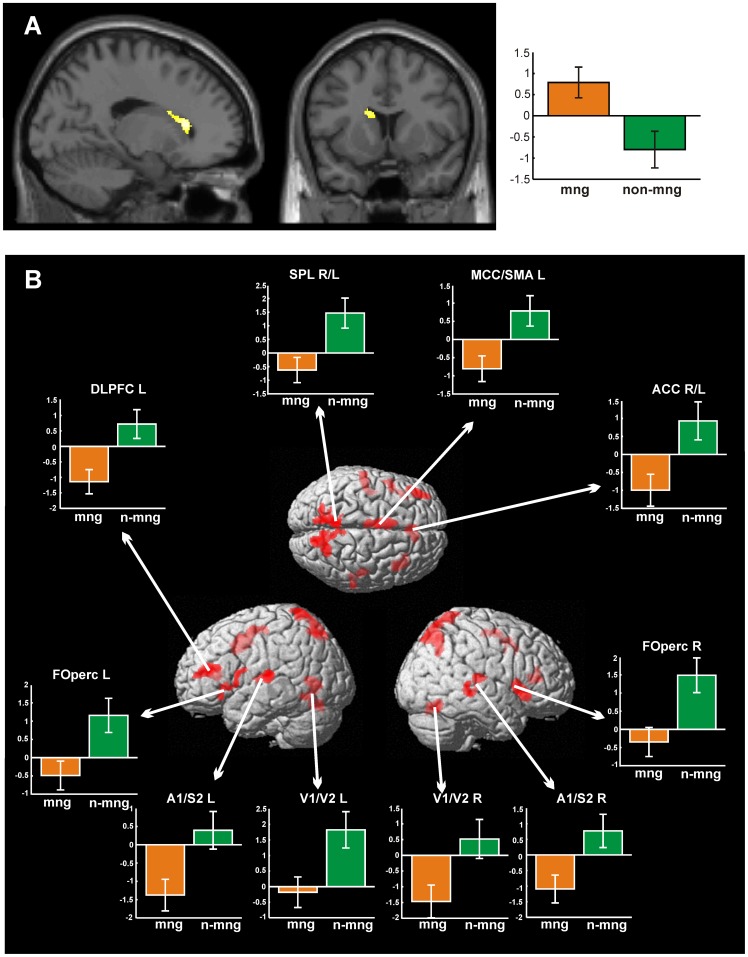
Significant differential effects of fMRI analysis (cluster-level corrected at p<0.0013). (A) Contrast managers (mng) > non-managers (n-mng). (B) Contrast non-managers > managers, projected onto sections and rendered surface of the MNI single subject template. Bar graphs provide mean-centred parameter estimates (i.e. strength of the BOLD response). Error bars provide 90% confidence intervals. *ACC* anterior cingulate cortex, *DLPFC* dorsolateral prefrontal cortex, *FOperc* frontal operculum, *MCC* midcingulate cortex, *SMA* supplementary motor area, *SPL* superior parietal lobule, *A1* primary auditory cortex, *S2* secondary somatosensory cortex, *V1/V2* primary/secondary visual cortex. R right, L left. Peak coordinates: Table 1.

A potential neural correlate for such a processing mechanism could be the basal ganglia system, particularly the dorsal striatum, which was found to be involved in categorization of stimuli based on rules and prior knowledge and thought to resemble intuitive fast processing [37], [38]. This particularly relates to automaticity learning which relies on the interaction of basal ganglia structures with cortical areas, especially within the frontal lobe [32]–[34], [38], [39]. We thus contrasted the fMRI data of the managers and non-managers during performance of the decision task. The managers indeed showed preponderant activation of the head of the caudate nucleus (Fig. 3A).

The head of the caudate was repeatedly found to be involved in habituation learning, which uses rules and prior knowledge [34], [37] to categorize incoming stimuli and select an adequate response [40]. It provides automaticity and online updating of information as a basis for rule-based learning [33], [38]. Recently, the role of the head of the caudate nucleus in the generation of automatic processing could be specified [33], [41]: Activation within the head of the caudate nucleus was found to increase after a short period of training. This was not correlated with performance accuracy after extensive training. This finding was interpreted as follows [41]: the head of the caudate nucleus should be responsible for initial rule learning in rule-guided behaviour as previously assumed [42]. Regarding the underlying mechanism, it was assumed that the caudate nucleus might train cortico-cortical connections which are the relevant processors of automated rule-guided behaviour after initial rule learning [43]. It thus acts as primer for activity within prefrontal cortex, to which it is strongly connected [44]. It was thus proposed that rule-based automaticity is initialized in the head of the caudate, proceeds to ventral premotor and later to dorsal premotor cortex as training is intensified [32]–[34], [39], [41]. The initial rule learning within the caudate nucleus might be responsible for finding the applicable rule for a given situation [39], [45], [46].

**Figure 4:**
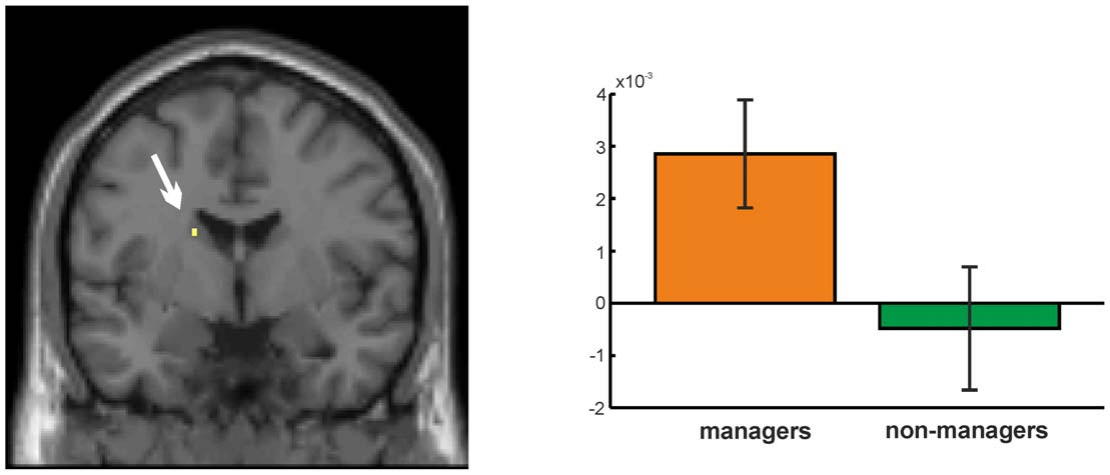
Significant time-related effect over the course of the experiment within the caudate nucleus (arrow, 3 voxels) for the managers as compared to the non-managers (p<0.001). Bar graphs provide mean-centred parameter estimates (i.e. strength of the BOLD response). Error bars provide 90% confidence intervals.

The literature on automated processing further suggests that activation within the caudate nucleus should show a time-related increase if equable decision scenarios are processed repeatedly [32], [33]. This should reflect a training effect, already after a short period of training. Increasing activity might additionally hint at the initialization of the rule-based automaticity [39], [41]–[43], [47]. It could therefore be assumed that the activation of the caudate nucleus which was found for the managers in the present study might increase over the time course of the experiment. This would further hint at an automated decision processing in the managers. Thus, an analysis of time-related effects within the caudate nucleus was carried out by implementing a linear time regressor on first-level analyses of all subjects which we tested for significant differences on the group level. Analysis of the hemodynamic response patterns revealed a significant correlation effect with time: for the managers as compared to the non-managers, activation within the head of the caudate nucleus was positively correlated with time (Fig. 4).

The findings of the present study thus suggest that behaviourally indicated differences in processing speed and number of decisions for a preferred category might find its neural correlate in the activation of the caudate nucleus in managers, which might reflect a more automated decision processing. The managers might have been using this rule-guided behaviour for fast categorization of the stimuli. This interpretation is supported by the reaction time analysis which showed that managers had shorter RTs as compared to non-managers when choosing a word of their preferred category. In dual-process theories, such a phenomenon was postulated: Very familiar concepts or ideas favour the use of a fast, automated processing system due to easy access to these concepts [5], [6]. Furthermore, the task of the present study was highly repetitive, providing the same kind of decision 540 times in a row. Subjects were able to use a rule which was generated internally by the subjects based on their value preference. Managers as expert decision-makers would seek to find a rule or heuristic on which they could base their decisions [6], [11]. According to previous studies, this phase of rule identification would involve the caudate nucleus [33], [39], [41]. Fully automated processing, after implementation of the relevant rule, should involve areas of the prefrontal cortex. This shift of activation from subcortical to cortical brain areas as a correlate for a shift from rule initiation to automated rule application typically requires a lot more repetitions than used in the present study [33], [39], [41]. How and when this shift happens in managers and if there are differences to non-managers, remains to be answered in future studies.

Contrarily, non-managers relative to managers recruited a distributed network of cortical areas, encompassing visual, superior parietal, temporal and frontal areas (Fig. 3B; Table 1).

**Table 1:** Coordinates of significant activations for main effects of fMRI analysis.

Functional/Macroanatomical label	Cytoarchitectonic label for cluster	T-stats	cluster size	x	y	z
**managers > non-managers**						
L head of caudate nucleus		4.62	313 voxels	−15	24	12
**non-managers > managers**						
L primary/secondary visual	17/18	4.03	580 voxels	27	−66	−20
L primary auditory/secondary somatosensory	TE1.0, OP 1	4.34	424 voxels	−51	−21	8
L posterior superior parietal	7A, 7P	4.54	1741 voxels	−10	−66	52
L frontal operculum		4.43	390 voxels	−34	26	−10
L dorsolateral prefrontal cortex		5.04	481 voxels	−40	40	16
L middle cingulate cortex		4.79	843 voxels	−8	9	42
L supplementary motor area	6	4.52		−8	−4	56
L anterior cingulated cortex		3.80	407 voxels	−3	36	16
R primary/secondary visual	17/18	4.33	606 voxels	−15	−65	3
R primary auditory/secondary somatosensory	TE1.0, TE1.1, OP 1	4.40	413 voxels	50	−26	12
R posterior superior parietal	7A, 7P	4.10	1741 voxels	12	−75	51
R frontal operculum		4.84	658 voxels	33	24	−8
R anterior cingulate cortex		4.52	407 voxels	8	34	10

Cytoarchitectonic labels refer to areas of the Jülich-Düsseldorf Cytoarchitectonic Atlas [48] as depicted in the SPM Anatomy toolbox [49]. Labels of the areas appear as published: 17/18 [50]; OP1 [51], [52]; 6 [53]; TE1.0, TE1.1 [54]; 7A, 7P [55], [56].

The way of processing in such a network is largely different from the processing ascribed to the caudate nucleus. It encompasses primary and secondary visual and auditory cortices representing stimulus-driven bottom-up processes. Activation of primary auditory cortex could have resulted from scanner noise. But recently, a different explanation was repeatedly assumed: Activation of a primary area of a sensory modality other than the main input modality (here: visual) might result from a priming effect, expecting additional incoming information [30], [31], [57]. These processes were complemented by top-down mechanisms accounting for regulation and gating of stimulus-driven processes [29], [58]. The superior parietal lobule e.g. processes the relevant stimulus (here, the visually presented words), being then forwarded to decision- and evaluation-related brain regions [31]. Brain regions associated with decision and evaluation processes were found within dorsolateral prefrontal cortex (DLPFC), anterior and midcingulate cortex (ACC, MCC). Activation within these regions was attributed to error and conflict detection, and weighing of alternatives [26], [27], [29], [58], [59]. Additional activation within the anterior insula at the border to the frontal operculum, was repeatedly found within such networks and assigned the role of cognitive control with regard to suppression of inadequate answers [28], [31], [57]. A comparable diversified network of cortical areas, involved in rule-based processing, was found in studies on the development of automaticity in cortical regions, namely from the first training session onward. Activation within this network did not change, even after extensive training, which was interpreted as not being responsible for the implementation of a rule-based automaticity [33], [39], [41].

The network activated by the non-managers during the repetitive decision task of the present study could thus likely be interpreted as such a primary processing network where bottom-up and top-down processes complement each other for processing of the incoming stimuli.

Taken together, these findings of the present study support our hypothesis that managers, being professionally used to decision-making, would rely on neural correlates which were previously linked to automated, categorization-based processing of stimuli in a situation of repetitively equable decisions. Contrarily, non-managers seem to involve a network of cortical areas which might reflect a step-wise processing of the stimuli via bottom-up (recognition of the stimuli) and top-down (selection of one stimulus) mechanisms. The present study hints at differential neural correlates for decision-making which lends support to the ideas from leadership and decision theory of managers as experts in decision-making taking a different approach as compared to non-managers [6], [11].

It might be speculated on the reasons for such differential neural correlates. It might be likely to assume that managers are trained by their job requirements with high pressure for decision-making [6], [11]. According to decision theory, this training would enable the managers to approach new situations by finding rules or heuristics to deal with the incoming information, having only limited resources for processing [2], [21]. But it is also possible that a manager is already equipped with this ability of rule-guided behaviour, and thus, becomes a manager only because he or she has this and other abilities. Based on the results of this study, this question could be approached in a longitudinal study design.

It has to be noted that the present study employed a paradigm of equable, abstract, and repetitive decisions. Such a situation might correspond to tasks of managers which occur repeatedly in their daily work experience. Contrarily, other tasks might need the managers to really focus their full attention on it to accurately carry them out. This might apply to tasks which require creative and new solutions. Similarly, managers might be good at identifying task priority. Thus, repetitive tasks, as implemented in the present study, could be completed quickly and without too much effort whereas others are more important, thus needing more attention and effort. Thus, the difference in neurobiological correlates of decision processing in managers as compared to non-managers could also reflect a more efficient way of allocating cognitive resources. Automatic processing therefore might not generally encompass decision situations per se, but rather selected decision processes such as equably repetitive decisions as in the present study. This might be elucidated in future studies.

### Reduced BOLD-response

**Figure 5:**
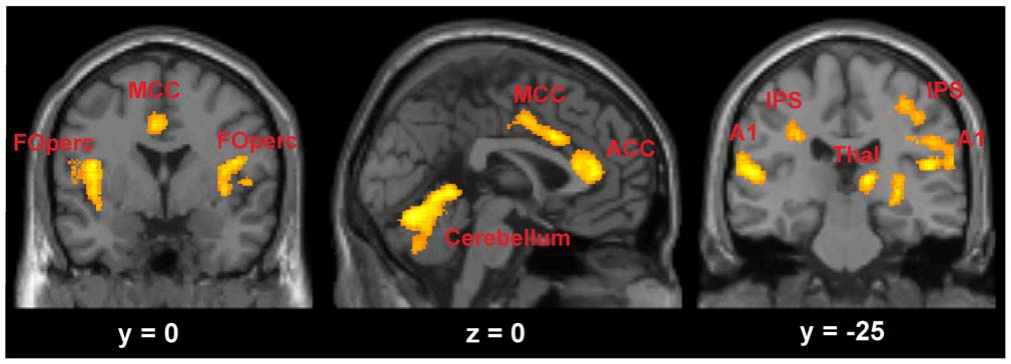
Areas of reduced hemodynamic response in managers. Activations projected onto coronal and sagittal sections of the MNI single subject template. *A1* primary auditory cortex, *ACC* anterior cingulate cortex, *FOperc* frontal operculum, *IPS* intraparietal sulcus, *MCC* midcingulate cortex, *Thal* thalamus.

The differential activation of cortical and subcortical areas between managers and non-managers not only resulted from activation of the cortical areas in non-managers, but coincided with a respective reduced BOLD-response of these same areas in managers. Analysis of reduced hemodynamic responses alone confirmed this pattern, showing additional significant reductions in the cerebellum and the right thalamus of the managers (Fig. 5).

The decision process of the managers was thus not only reflected by preponderant striatal activation. It was rather combined with reduced activation or active inhibition in cortical areas which, in interaction with the striatum, might support the role of the striatum in automated, fast online-updating of information during a decision process [37], [38].

Reduced BOLD-response during task execution has been repeatedly reported, e.g. during attentional shifts to external cues [60]–[62]. This was complemented by findings that adequate preparation for task execution requires activation of task-relevant areas [63], [64]. Additionally, task-irrelevant areas need to be suppressed for successful performance of a task [65]. This view was supported by findings in patients suffering from traumatic brain injury which had difficulty to sustain endogenously driven attention during a task [66], [67].

Discussing deactivation patterns or reduced BOLD-responses during fMRI studies necessarily needs to consider its potentially related meaning. There is still a controversy due to limited evidence on how reduced BOLD-response is related to the underlying neuronal activity [68], [69]. Shmuel et al. [70] could show that reduced spiking activity of neurons was spatially correlated with a reduction of the BOLD-response. Conversely, Devor et al. [71] demonstrated that hyperpolarization of neurons and consecutive deoxygenation coincides with vasoconstriction. Thus, they proposed that the inhibitory activity of the neurons was responsible for the vasoconstriction [72], in contradiction to the former model that introduced reduced spiking activity as the responsible factor [70]. Consequently, a reduced BOLD-response in fMRI could indicate either reduced activation of an area or active inhibition of this area [68].

With regard to the results of the present study, it is thus possible that the managers actively inhibited the network of cortical, cerebellar, and thalamic regions or that these areas were not as active as the predominantly activated head of the caudate nucleus. Reduced activation within this network might indicate that all these areas, together with the head of the caudate, were involved in the decision process. But the head of the caudate was predominantly activated, potentially in order to initialize rule-guided behaviour as described in former studies [39], [41], [43], [47]. Since the cortical areas were repeatedly found to be involved in decision-making and proposed to be essential for further automation of the decision process [34], [43], it seems likely to assume that this model of less activation in the cortex also describes the results within the managers.

Active inhibition of the areas in the managers would go beyond this explanation. This would suggest that the managers actively suppressed cortical and cerebellar regions, with resources concentrated on activation of the caudate nucleus as promoter for automaticity initiation. With the thalamus being the gating structure for incoming sensory information to cortical areas [73], managers not only down-regulated the cortical targets, but also the relay station to regulate input to the cortex.

Both explanations provide further insight on the complex organisation of the decision process, especially with regard to the potential automation of reasoning in the managers. Which interpretation of the BOLD-response is adequate for explaining the observed effects in the present study remains tentative until more basic evidence on the physiological meaning of a reduced BOLD-response is available.

### Conclusions and outlook

Our results provide first evidence for two dissociated, but interacting decision processing systems in expert and non-expert decision-makers in an abstract, repetitive decision scenario. The generalization of these findings to other, concrete decision-making tasks with real-world scenarios needs to be elucidated. Moreover, the reason for this dissociation between managers and non-managers requires additional research: The dissociation might reflect an adaptation effect due to professional requirements of the job as a manager, i.e. decision-maker [21]. Alternatively, it might also be caused by different personality or behavioural traits of persons, suggesting this decision processing mechanism to be a prerequisite to become a manager.

## Materials and Methods

### Ethics statement

All subjects gave written informed consent to the study protocol as approved by the local ethics committee of the Rheinisch-Westfaelische Technische Hochschule (RWTH) Aachen University.

### Participants

44 business managers (mean age ± SD  = 44.34±6.48, 22 males, 22 females) participated in the experiment, of which 35 (18 males, 17 females) were included in further analysis based on their choice profile (cf. Results). Only managers with at least five directly-reporting subordinates were included in the experiment to assure that all managers had experience in leadership of employees. Managers who participated in the present study had management responsibilities for a median number of 15 employees (range 5–3000 employees, skewness 4.9, kurtosis 25.6). Managers were all office managers, coming from different companies to cover a wide range of manager personalities with different backgrounds of corporate culture. These included e.g. banks, consulting agencies, department stores, IT companies, or research institutions. They worked as group leaders, department managers or directors in marketing, human resources, controlling, sale etc. Based on management theory, it is assumed that all these managers have a high need for making decisions in different work settings. In the present study, one specific type of decision making, i.e. equably repetitive decisions, was tested as one type of decisions occurring in daily work life (cf. next section).

As a control group, a total number of 82 subjects with no leadership experience participated. Out of these, 35 (mean age ± SD  = 40.37±10.80, 23 males, 12 females) were included in the present analysis based on their choice performance during accomplishment of the task in the scanner (see below), thus serving as controls for the managers. The performance of the controls should match performance of the managers to eliminate a potentially confounding factor from further analysis.

All participants had no history of neurological or psychiatric disease and normal or corrected to normal vision.

### Experimental design, stimuli, and stimulus presentation

Each participant performed a functional magnetic resonance imaging (fMRI) experiment, using a forced-choice paradigm on abstract value words [35]. Words were generated based on psychological value theories [36], [74]–[75], with a main distinction between individualistic and collectivistic value words (Table 2).

**Table 2:** Stimulus words used for the fMRI paradigm (two categories, 18 words each).

**collectivistic**	‘Zusammengehörigkeit’	‘Sicherheit’	‘Menschlichkeit’
	*togetherness*	*safety*	*humanity*
	‘Geborgenheit’	‘Sorgfalt’	‘Harmonie’
	*protection*	*diligence*	*harmony*
	‘Familie’	‘Loyalität’	‘Gemeinschaft’
	*family*	*loyalty*	*community*
	‘Tradition’	‘Verantwortung’	‘Teamfähigkeit’
	*tradition*	*responsibility*	*teamwork*
	‘Zusammenhalt’	‘Gerechtigkeit’	‘Konvention’
	*solidarity*	*fairness*	*convention*
	‘Beständigkeit’	‘Maßstäbe’	‘Geselligkeit’
	*constancy*	*standards*	*sociability*
**individualistic**	‘Spaß’	‘Erfolg’	‘Flexibilität’
	*fun*	*success*	*flexibility*
	‘Kreativität’	‘Selbständigkeit’	‘Wertschätzung’
	*creativity*	*autonomy*	*esteem*
	‘Macht’	‘Kompetenz’	‘Unabhängigkeit’
	*power*	*competence*	*independence*
	‘Status’	‘Leistung’	‘Nachsicht’
	*status*	*performance*	*indulgence*
	‘Respekt	‘Risikobereitschaft’	‘Hingabe’
	*respect*	*risk-taking*	*commitment*
	‘Herausforderung’	‘Zielstrebigkeit’	‘Selbstentfaltung’
	*challenge*	*determination*	*self-development*

Stimulus words in the table as the original German word (in single quotation marks) and as English translation beneath (in italics). Words as used in a previous study [35], based on value theories [36], [74]–[76].

This task was chosen for the present study since it provided an abstract set of stimuli, independent of a concrete real-world situation, thus being much more simplified than a real scenario. The usage of abstract moral values forced the subjects to decide based on their general value preference. They thus had some prior knowledge about the different categories of moral values and could choose in accordance to their own preferences [36], [74]–[76]. The task was demanding to require constant attention and conscious decision-making in every single trial. Furthermore, the task simulated a situation of repetitive and equable decisions as one type of decision situations in real life.

For each of the two categories (individualistic and collectivistic), 18 words were generated. Due to ambivalent meaning of original value words, stimulus words were checked for accuracy and selectiveness using the German Duden glossary of synonyms [77] and affirmed by speech and language therapists of the Neurolinguistics Department of the RWTH Aachen University. Furthermore, as German language is case sensitive, only nouns had to be generated which were not adjective- or verb-derived to assure consistency in syntactic word category [35].

**Figure 6:**
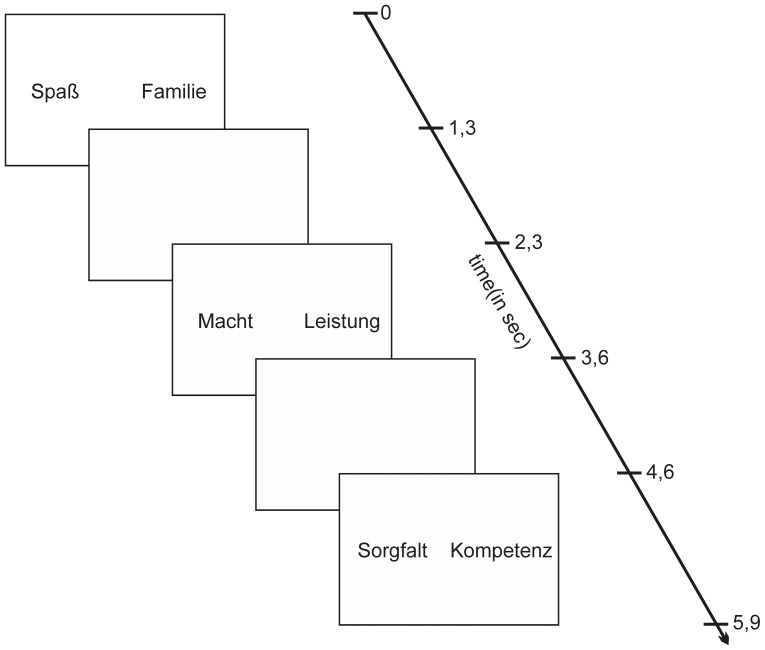
Exemplary sketch of the paradigm design. Word pairs are shown as the original German stimulus words (for translations, cf. Table 2).

Two words belonging to different value categories were presented simultaneously on either side of a screen (word pair, Fig. 6). With two word categories, a total number of 36 words were at disposal. Every word from each category was combined with each other word from the same or the other category, providing a total number of 324 trials. Interleaved and randomized across participants, word pairs consisting of either two individualistic (108 trials) or two collectivistic words (108 trials) were presented as catch trials to assure equably stable attention over all trials and the need to decide on each word pair independently. The value words as used in the present paradigm could further be subdivided into subcategories of different levels of complexity and close similarity in value meaning [74]. Thus, these words were not combined with each other. In total, 540 word pairs were presented. It has to be noted that differences in neural correlates could only be observed for processing of the main categories ‘individualistic’ and ‘collectivistic’, without any further differentiation for any of the value subcategories [35]. Therefore, the present analysis considered this main distinction as potential influencing factors when investigating the neural correlates of decision strategies in managers and non-managers.

Prior to scanning subjects were instructed to spontaneously select the most appealing word in each presented word pair. They were only informed on the general design of the study, i.e. that pairs of words would be presented in a rapid sequence, leaving them no time to carefully think about their choice. Participants were naive about the intention of the study to assure impartiality. They were instructed to report their choice by button press, using the right index finger when choosing the word on the right side of the screen and the left index finger for the word on the left side of the screen. After scanning, subjects were debriefed. They were asked to provide a short appraisal of how they experienced the choice situations during the experiment.

Words were presented as written strings in Helvetica font at 48 pts, with the two words in each word pair being located equally distant from the centre of the screen. Over all 540 trials, each word appeared 30 times with 50% appearance on the left and 50% on the right side of the screen to avoid habituation effects or preferences of the subjects for one side of the screen. Word pairs occurred in randomized order, with different randomization for every participant.

Subjects saw the stimuli (presented by Presentation®; Neurobehavioural Systems, Albany, USA), which were back-projected onto a screen at the back wall of the scanner room, via an angled mirror suspended from the head coil.

The paradigm was implemented as a modified event-related design. The whole experiment lasted about 22 minutes. Each presentation of a word pair lasted 1.3 seconds, followed by a blank screen period of 1 second, giving a stimulus onset asynchrony (SOA) of 2.3 seconds. The short presentation time of the stimuli was chosen to avoid social desirability biases which might occur if subjects are given enough time to rethink their answer [36], [74]–[76]. Since linear additivity and general independence can be assumed for trials with onsets of at least 1 second apart [78], [79], the hemodynamic response of each voxel in the brain can be decomposed for analysis of the fMRI data. Jittering of the trials in relation to the repetition time (TR) of the scanner (cf. next paragraph) was constructed by implementation of a temporal jitter using distributed sampling [80], [81]. Thus, trial durations (2.3 seconds) were slightly shorter than acquisition of one complete MR dataset of the brain. Therefore, the same voxel was scanned at different time points during the experiment. This procedure was chosen to assure equally short presentation times for each word pair.

### Data acquisition of functional and anatomical magnetic resonance images

The experiment was run on a 3T Siemens Tim-TRIO MR-scanner (Erlangen, Germany), using a standard birdcage head coil for data acquisition with foam paddings to reduce head motion. Functional imaging data were acquired from the whole brain by using a gradient-echo echoplanar imaging (EPI) sequence for blood-oxygen-level-dependent (BOLD) contrast (parameters: echo time (TE)  = 30 ms, repetition time (TR)  = 2.5 s, flip angle  = 90°, 41 axial slices, 3 mm slice thickness, slice distance 10%, field of view (FoV)  = 200×200 mm^2^, giving an in-plane resolution of 3×3 mm^2^). After the experimental EPI runs, a high-resolution T1-weighted anatomical image was obtained for later normalisation of the EPI data into the standard reference space of the Montreal Neurological Institute (MNI), using a 3D-MPRAGE sequence (parameters: 176 slices, TR  = 2.25 s, TE  = 3.03 ms, FoV  = 256×256 mm^2^, flip angle  = 9°, final voxel resolution: 1×1×1 mm^3^).

### Functional image analysis

Data were processed using MATLAB 7 (The Mathworks Inc., Natick, USA) and the SPM5 software package (Wellcome Department of Imaging Neuroscience, London, UK, http://www.fil.ion.ucl.ac.uk). Pre-processing of the data included realignment, segmentation using the unified segmentation approach [82], normalisation to the MNI single subject template [83], and spatial smoothing with an 8 mm FWHM Gaussian kernel.

The aim of the present experiment was studying the general decision processing in managers vs. non-managers. Thus, for the analysis of the brain activation data, all trials were collapsed into one condition which reflected the decision process during the experiment. This enabled general modelling of the relevant decision processes which were needed to process all presented decision situations, and thus detection of neural correlates for potentially different decision strategies in managers and non-managers. Separate analyses for either trial type (i.e. individualistic, collectivistic) were carried out as well, yielding no significant results. Trials for which subjects failed to answer within the time frame of 2.3 seconds were discarded from further analysis. Trials were analysed in a modified event-related fashion, aiming at optimally modelling the relevant time period of each event, encompassing attainment of the stimulus, cognitive processing and decision making [84]. For each event, the duration was set individually according to the subject's response time at the time of button press. Subjects were allowed to respond at any time point between presentation of a stimulus and presentation of the next stimulus (2.3 seconds response timeframe). Subject's response did not influence presentation time of a word pair which was always 1.3 seconds.

Additionally, a time regressor was added for each subject to model the course of the hemodynamic response during the decision process. This allowed assessing training effects due to longer experience with the paradigm (equably repetitive decisions on abstract word pairs).

The event-related block functions for each category were convolved with the hemodynamic response function (HRF) and its first derivative for a more flexible fit to the data. Assumptions about single events based on the emerging total HRF could be made assuming additive effects according to the Linearity Theory for event-related designs with stimulus-onset asynchrony of around 1 second [85], [86]. For each trial category, a baseline contrast with the implicit baseline as implemented in SPM was used, where the implicit baseline consisted of all blank screen intervals between consecutive trials. For response times longer than the presentation of the stimulus, only the rest of the blank screen period was included in the implicit baseline.

On the second level for a group analysis, individual contrast images of both trial categories were entered into a random-effects analysis. For analysis, a full factorial design was implemented with factors ‘subject’ (necessary to model repetition as a within-subject factor), and ‘group’ (either managers or non-managers), using t-tests to assess differences between the two groups. Coordinates are reported in standard MNI space [87].

### Statistical analysis of neuropsychological and behavioural data

Neuropsychological and behavioural data were analysed using SPSS Statistics 19 for Windows (IBM Corp., Somers, NY, USA).

Subjects' performance during the fMRI experiment, i.e. the decisions made by the subjects for either word in each word pair, was tested by means of a two-step cluster-analysis. The aim was to identify subgroups of either individualistic or collectivistic value preference [35]. Decisions of each subject during the fMRI experiment were categorized into either belonging to the individualistic or the collectivistic word category. For each participant, the number of choices for either category entered the analysis (standardized for further analysis during clustering, Bonferroni-corrected for multiple comparisons; parameters: 15 clusters, log-likelihood distance estimation, clustering criterion: Akaike's information criterion, no noise-handling for outlier treatment, initial distance change threshold  = 0, 8 branches per leaf node, 3 depth levels). As additional neuropsychological testing, each participant underwent IQ testing using the short form (part 1) of the culture-free intelligence test CFT-20 [88].
